# Hepatocellular Carcinoma Recognition from Ultrasound Images Using Combinations of Conventional and Deep Learning Techniques

**DOI:** 10.3390/s23052520

**Published:** 2023-02-24

**Authors:** Delia-Alexandrina Mitrea, Raluca Brehar, Sergiu Nedevschi, Monica Lupsor-Platon, Mihai Socaciu, Radu Badea

**Affiliations:** 1 Department of Computer Science, Faculty of Automation and Computer Science, Technical University of Cluj-Napoca, 400114 Cluj-Napoca, Romania; 2 Department of Medical Imaging, “Iuliu Hatieganu” University of Medicine and Pharmacy, 400347 Cluj-Napoca, Romania; 3 “Prof. Dr. O. Fodor” Regional Institute of Gastroenterology and Hepatology, 400162 Cluj-Napoca, Romania

**Keywords:** convolutional neural networks (CNN), conventional machine learning (CML), advanced texture analysis methods, combination techniques, classification performance, hepatocellular carcinoma (HCC), ultrasound images

## Abstract

Hepatocellular Carcinoma (HCC) is the most frequent malignant liver tumor and the third cause of cancer-related deaths worldwide. For many years, the golden standard for HCC diagnosis has been the needle biopsy, which is invasive and carries risks. Computerized methods are due to achieve a noninvasive, accurate HCC detection process based on medical images. We developed image analysis and recognition methods to perform automatic and computer-aided diagnosis of HCC. Conventional approaches that combined advanced texture analysis, mainly based on Generalized Co-occurrence Matrices (GCM) with traditional classifiers, as well as deep learning approaches based on Convolutional Neural Networks (CNN) and Stacked Denoising Autoencoders (SAE), were involved in our research. The best accuracy of 91% was achieved for B-mode ultrasound images through CNN by our research group. In this work, we combined the classical approaches with CNN techniques, within B-mode ultrasound images. The combination was performed at the classifier level. The CNN features obtained at the output of various convolution layers were combined with powerful textural features, then supervised classifiers were employed. The experiments were conducted on two datasets, acquired with different ultrasound machines. The best performance, above 98%, overpassed our previous results, as well as representative state-of-the-art results.

## 1. Introduction

Cancer is a severe affection which seriously threatens human health and sometimes leads to death. HCC is one of the biggest health problems in gastroenterology. It represents the most frequent primary cancer of the liver, the fourth most frequent cancer in men and the seventh most frequent cancer in women. It is also the third most frequent cancer-related cause of death, after lung cancer and colorectal cancer [1]. In the majority of cases, HCC evolves from cirrhosis, after a liver parenchyma restructuring phase at the end of which dysplastic nodules result, which can transform into HCC [2]. The presence of cirrhosis makes both the diagnosis and the treatment harder to perform: the presence of an underlying nodular pattern in cirrhosis makes the detection of the HCC nodular forms a daunting task. For many years, the golden standard for HCC diagnosis has been the needle biopsy, which is invasive and also raises risks, as it could generate infections and can lead to the spread of the tumor through the human body, respectively. However, the only viable way of detecting early HCC considered nowadays is medical imaging, because the clinical and biological markers lack sensitivity in this case. One way of diagnosing HCC is by conducting vascular contrast-enhanced imaging studies, which rely on specific patterns of contrast enhancement in malignant tumors due to the effects of oncogenesis on local vascularity. The performance of these methods has gone so far that, in most cases, percutaneous biopsy is not even recommended for the definitive diagnosis [3]. However, of the three imaging methods, namely computed tomography (CT), magnetic resonance imaging (MRI) and ultrasonography (US), only the latter can be used as a screening option among cirrhotic patients, due to lack of availability for large populations (for both CT and MRI) and high radiation burden for CT, respectively. The B-mode (grayscale) ultrasound images are two-dimensional images that render the tissues and structures of interest as points of variable brightness. The ultrasound waves, generated by the transducer, are reflected by these tissues and structures, so the pixel values are related to the intensity of this reflection. These values contain important information concerning the nature of the corresponding tissues and structures. The resulting complex textures inside the tumors, different from those in the cirrhotic liver, might hold the answer for a better detection rate through computer analysis. Thus, noninvasive, computerized methods are due for detecting HCC as early and accurately as possible, revealing subtle aspects upon the tissue structure.

**Figure 1:**
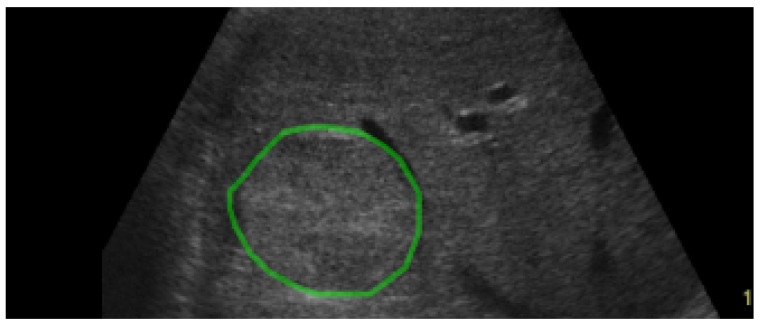
The visual aspect of HCC in ultrasound images: the HCC contour is marked with green.

In ultrasound images, early HCC appears as a small, usually hypoechogenic nodule, without a visible capsule, having 2–3 cm in diameter. In more advanced stages, HCC increases in size, develops a hyperechogenic capsule, invades liver vessels and may present various visual US aspects, usually becoming overall hyperechogenic and heterogeneous due to the interleave of multiple tissue types, such as normal liver, fatty cells, active growth tissue or necrosis. Thus, in some situations, the increased echogenicity, heterogeneity and delimiting capsule of HCC are not that obvious. As both HCC and cirrhotic parenchyma represent forms of tissue restructuring, in many situations they can hardly be differentiated by the human eye [2]. An eloquent example is provided in Figure 1.

In the current approach, we developed and assessed appropriate methods for combining conventional and deep learning techniques, the final purpose being to improve automatic HCC recognition based on medical images, with respect to the already existing results. These combinations were performed at classifier level. The values of the textural features were fused in various manners with those of the features obtained at the outputs of different layers of representative CNN architectures, then provided to a single supervised classifier. Appropriate dimensionality reduction methods, such as feature selection techniques and Kernel Principal Component Analysis (KPCA), were applied in this context, with the results being carefully analyzed. The relevance of the considered features and the correlations between the textural and deep learning features, as well as the corresponding medical significance, were discussed. The experiments were performed on two datasets, acquired with two different ultrasound machines. These datasets contained regions of interest corresponding to the HCC tumor and the cirrhotic parenchyma on which HCC had evolved, respectively.

In the context of our previous research, we developed and experimented with both conventional and deep learning methods for HCC recognition from ultrasound images. Regarding the conventional techniques, we defined the textural imagistic model of HCC, as described in [4]. Original and advanced texture analysis methods were developed and experimented with in this context, most of them based on Generalized Cooccurrence Matrices (GCM) of second and superior order [5]. The values of the most relevant textural features were provided at the entrances of powerful classifiers, such as Support Vector Machines (SVM), Multilayer Perceptron (MLP) and Random Forest (RF), along with AdaBoost combined with the C4.5 method for decision trees. The maximum accuracy was 84.09% when differentiated between HCC and the cirrhotic parenchyma, respectively, and it was 88.41% when differentiating between HCC and the hemangioma benign tumor [5]. As for the deep learning methods, we developed and assessed multiple techniques, mainly based on CNNs [6,7] but also on Stacked Denoising Autoencoders (SAE) [8]. A maximum classification accuracy of 91% resulted from the same dataset in the case when differentiating HCC from the cirrhotic parenchyma on which it had evolved.

### 1.1. The State of the Art

Regarding other representative methods belonging to the state of the art of the domain, conventional techniques combining texture analysis methods with traditional classifiers were widely applied previously for performing the automatic diagnosis of tumors and other affections within medical images [9,10,11]. Recently, the deep learning methods were extensively employed in the field of computer vision, particularly in medical imaging, leading to successful results. The CNNs demonstrated their value in both supervised and unsupervised approaches, such as those presented in [12,13,14]. Relevant approaches, referring to the automatic diagnosis of liver tumors, as well as of other affections (cirrhosis, which precedes liver cancer and other type of tumors), are described below.

#### 1.1.1. Existing Approaches Targeting the Automatic Recognition of Liver Tumors from Medical Images

The conventional methods that combine texture analysis with supervised traditional classifiers were previously used in order to perform liver tumor recognition within medical images. The textural parameters were first employed by Raeth in [9] for differentiating between normal liver, diffuse liver diseases and malignant liver tumors from ultrasound images. Various types of textural features were employed for this purpose, such as those derived from the intensity histograms, those obtained from the run-length matrix, edge- and gradient-based features, the second-order Gray-Level Co-occurrence Matrix (GLCM) matrix and the associated Haralick features, co-occurrence matrices based on edge orientations and other gradient features and features derived through the Fourier transform, as well as parameters referring to speckle noise. All these features were provided to a decision-trees-based classifier that differentiated among pairs of classes, such as tumoral and nontumoral tissue, fatty and cirrhotic liver, normal liver tissue and hepatitis. In another similar approach, the run-length matrix and the corresponding parameters, in combination with the Haralick features resulted from GLCM, were experimented with in conjunction with ANN classifiers, SVM and Fisher Linear Discriminants (FLD), targeting the automatic diagnosis of the liver lesions within ultrasound images [15]. The ANN classifier, having a recognition rate close to 100%, overpassed the FLD technique, which yielded a classification accuracy of 79.6%. The Wavelet transform, applied in a recursive manner [10], as well as in combination with fractal features [16], was also involved in the ultrasound-images-based recognition of HCC. In the first case, a recognition rate of 90% resulted from employing an ANN classifier, while in the second case, an accuracy of 92% was achieved through the same type of classifier. More recent approaches that performed the automatic recognition of the liver tumors were based on CNNs. A relevant methodology that proposed a deep learning model for HCC automatic diagnosis was presented in [17]. The authors employed a ResNet18 CNN pretrained with the ImageNet dataset, the training being then refined using hematoxylinand eosin-stained pathological slides gathered from 592 HCC patients. At the end, a slide-level accuracy of 98.77% resulted. Another method, aiming for liver lesion segmentation from CT images, was presented in [18]. A specific CNN was trained by using image patches obtained from 67 tumors of 21 patients, in order to perform voxel classification as part of the segmentation process. These patches contained both tumor and healthy liver tissue. A success rate of 95.4% and an average overlap error of 16.3% resulted.

#### 1.1.2. Existing Approaches That Employ Deep Learning for the Recognition of Other Affections Based on Medical Images

The estimation of the cirrhosis severity grades from 2D shear wave elastographic images for patients affected by chronic B-type hepatitis was performed in [12], where a CNN containing four convolution layers and a single fully connected layer was employed. The training set consisted of 1990 images corresponding to 398 cases. At the end, an AuC of 0.85 was achieved. A relevant approach involving CNN-based techniques was presented in [19]. The purpose was to detect breast tumor structures from ultrasound images using a CNN-based method called Single Shot MultiBox Detector (SSD). The corresponding dataset comprised 579 benign and 464 malignant breast lesion cases. The proposed method yielded better performance in terms of precision and recall as compared with the other existing state-of-the-art methods. A Deep Convolutional Neural Network (DCNN) was implemented in [20] for detecting incipient lung cancer from CT images. The experimental dataset consisted of 62,492 regions of interest extracted from 40,772 nodules, as well as of 21,720 non-nodules belonging to the Lung Image Database Consortium (LIDC) data store. A maximum classification accuracy of 86.4% resulted for this methodology. In the latest years, more complex approaches were developed for achieving an increased pathology recognition performance. The combination of multiple image modalities was analyzed in several studies, such as [21,22]. B-mode ultrasound images were combined with CEUS images through CNN-based techniques in order to automatically recognize breast tumors [21], and histological and immunohistochemical image data were fused in [22] through a CNN-based methodology for breast cancer diagnosis. Other approaches combined multiple types of deep learning features. In [23], aiming to detect breast cancer within histopathology images, the authors combined the deep learning features provided by the VGG16, InceptionV3 and ResNet50 architectures, the concatenated features being provided to a VirNet model, which performed the final feature fusion and classification. In the approach described in [24], the authors combined the deep learning features provided by the ResNet50 and DenseNet201 architectures for performing brain tumor classification. After a feature selection process, the relevant features were fused using a serial approach and provided to an SVM classifier that provided an 87.8% classification accuracy.

#### 1.1.3. Existing Approaches That Combine Conventional and Deep Learning Techniques

Moreover, the combination between conventional and deep learning techniques was exploited in the domain for further improvement of the classification performance. As conventional features, radiomic features (intensity and texture), as well as other types of handcrafted features such as the Histogram of Oriented Gradients (HOG), were considered. These types of approaches are analyzed in the next paragraphs, mainly referring to the medical imaging domain. A relevant approach regarding classifier-level fusion is described in [25], where the authors studied the combination of deep learning and radiomic features for assessing PD-L1 expression level via preoperative MRI in HCC cases. An extended set of radiomic features were derived from the Volumes of Interest (VOI) using the *Pyradiomics* tool. These features included textural features, intensity features (first-order statistics) and geometric (shape) characteristics. The textural features comprised GLCM features, Gray-Level Size Zone Matrix (GLSZM) features, Neighboring Gray Tone Difference Matrix (NGTDM) features, Gray-Level Run-Length Matrix (GLRLM) features and Gray-Level Dependence Matrix (GLDM) features, respectively. In addition, the derived images were determined by applying eight types of image filters: gradient, wavelet, square, square root, logarithm, exponential, Laplacian of Gaussian (LoG) and 3D Local Binary Pattern (LBP-3D). The intensity and textural features were determined on the derived images as well. In order to obtain the deep learning features, an original 3D CNN architecture was developed, consisting of two 3D convolution layers and two fully connected layers. The deep learning features were extracted from the output of the first fully connected layer after applying the rectified linear unit (ReLu) activation function. The radiomic features were concatenated with the deep learning features, then a normalization procedure was applied, a redundant feature removal process was employed and the result was provided to a supervised classifier of the Support Vector Machine–Recursive Feature Elimination (SVM-RFE) type, which also performed feature selection. For eliminating the redundant features, the Pearson Correlation Coefficient (PCC) was implemented. The experiments were performed on an HCC dataset corresponding to 103 patients. The correlation between the relevant radiomic features and the PD-L1 expression level was also established, the considered classes being correlated with the PD-L1 expression level as well. An accuracy of 88.7% and a precision of 94.8% resulted, regarding the prediction of the PD-L1 expression level. Aiming to improve the state-of-the-art performance concerning the prediction of lymph node metastasis in head and neck cancer, in [26], the authors combined conventional radiomic and deep learning features resulted from CT and Positron Emission Tomography (PET) images. A new many-objective radiomics model (MO-radiomics) was designed for extracting valuable radiomic features, and an original 3D CNN architecture that fully utilized spatial and contextual information was employed for yielding the deep learning features. The MO-radiomics model consisted of textural features and intensity features (first-order statistics), respectively, as well as geometric features. The textural feature set comprised 3D GLCM features, a total of 257 features being finally extracted from the CT and PET images. Then, the SVM method was employed in order to build a predictive model, and an optimization problem was solved for selecting the final feature set and model parameters. Concerning the 3D CNN model, the corresponding architecture consisted of 12 convolutional layers, 2 max-pooling layers and 2 fully connected layers. The conventional and deep learning features were fused through an *evidential reasoning* method. The performance of this hybrid method was assessed for classifying normal, suspicious and lymph node metastasis. The proposed hybrid methodology finally led to a 92% accuracy, which overpassed the 79% accuracy of the conventional methods of the proposed 3D CNN. Another approach was illustrated in [27], where the authors fused deep learning features with radiomic features for predicting malignant lung nodules from CT images. The deep learning features provided by VGG-type CNNs, as well as by originally designed CNNs, were fused with classical radiomic features, including size, shape, GLCM, Laws and Wavelet features. A Symmetric Uncertainty technique was employed to select relevant attributes from both deep learning and conventional feature sets, then the fused set was given as input to an RF classifier. The best accuracy of 76.79% was obtained when employing VGG-type CNNs. The methodology presented in [28] also demonstrated that the combination of deep learning and radiomic features led to the highest performance in lung cancer survival prediction. Thus, several pretrained CNNs were adopted to obtain deep features from 40 contrast-enhanced CT images, representing non-small-cell adenocarcinoma lung cancer, these being combined with handcrafted features. The deep learning features were obtained before and after the last Rectified Linear Unit (ReLU) layer. Thereafter, multiple supervised classifiers, receiving relevant features at their inputs, were compared, achieving a maximum accuracy of 90% and a maximum AUC of 93.5%, respectively.

### 1.2. Contributions

According to the previous paragraph, there are many approaches that combine conventional features with deep learning features, demonstrating classification performance improvements. However, there are no relevant approaches that combine conventional and deep learning features for performing HCC automatic diagnosis within ultrasound images. We studied this possibility in our current work, aiming to further improve the accuracy of noninvasive HCC automatic diagnosis. Thus, the contributions of our current research are the following: (1) We combined conventional machine learning methods, involving advanced texture analysis methods with deep learning classifiers based on CNNs, to automatically recognize HCC tumors within B-mode ultrasound images. (2) We performed classifier-level fusion by experimenting with combination schemes involving various dimensionality reduction methods for obtaining the most valuable information from the whole data, such as relevant features or the main variation modes. An increase in the computational efficiency was an objective as well. Thus, we considered dimensionality reduction methods from both categories of feature selection and feature extraction techniques, the KPCA technique being taken into account for the second category. Regarding the feature selection techniques, we considered both classical techniques, as well bio-inspired approaches based on Particle Swarm Optimization (PSO). We mined for possible correlations among the textural and deep learning features. (3) As for the conventional techniques, we considered a large variety of textural features based on both classical and advanced original texture analysis methods, such as the superior-order Generalized Co-occurrence Matrices (GCM). We also considered multiresolution features, achieved after recursively applying the Wavelet transform. (4) Regarding the CNN-based techniques, we considered existing, representative deep learning architectures, as well as new architectures, improved by the authors in an original manner, starting from the standard architectures. (5) We updated the definition for the textural imagistic model of HCC [4], considering the combinations between the conventional and deep learning techniques, with appropriate experiments being performed. (6) We performed the experiments on two datasets of B-mode ultrasound images, constituted by the authors, acquired with two different ultrasound machines.

## 2. Materials and Methods

### 2.1. Description of the Experimental Datasets

For performing reliable experiments, two HCC B-mode ultrasound image datasets were exploited. The first one, denoted by *GE7*, contained B-mode ultrasound images corresponding to 200 HCC cases, acquired with a Logiq 7 (General Electric Healthcare, Chicago, IL, USA) ultrasound machine, under the same settings: frequency of 5.5 MHz, gain of 78, depth of 16 cm and a Dynamic Range (DR) of 111. The second dataset, denoted by *GE9*, consisted of B-mode ultrasound images belonging to 96 patients affected by HCC, acquired through a newer, Logiq 9 (General Electric Healthcare, Chicago, IL, USA) ultrasound machine, using the following set-up parameters: frequency of 6.0 MHz, gain of 58, depth of 16 cm and a DR of 69. These images were gathered by medical specialists at the 3rd Medical Clinic in Cluj-Napoca, respectively, at the “Octavian Fodor” Regional Institute of Gastroenterology and Hepatology in Cluj-Napoca. All the patients included in this study underwent biopsies for diagnostic confirmation. For each patient, multiple images were considered, corresponding to various orientations of the ultrasound transducer. Two classes were considered for differentiation in our study, these being HCC and the cirrhotic parenchyma on which HCC had evolved (denoted by PAR). The two classes were employed, as they are visually similar in many situations, it also being known that the HCC tumor usually evolves on cirrhotic parenchyma. This focus was suggested by the experienced radiologists, so no normal (healthy) cases were included in this study. The GE7 dataset included HCC tumors in various evolution phases. For this dataset, acquired previously, rectangular regions of interest having 50 × 50 pixels in size were manually selected by the specialized physicians inside the HCC tumor or on the cirrhotic parenchyma using a specific application implemented by the authors. The GE9 dataset comprised mostly advanced-stage HCC tumors. For this dataset, recently gathered, the HCC structures were manually delineated by the medical specialists, using the VGG Image Annotator (VIA) [29] application. Through the VIA interface, the specialists delimited the tumoral region through a polygon. According to these delimitations, rectangular regions of interest (patches) having 56 × 56 pixels in size were automatically extracted from the tumoral regions, respectively, from the cirrhotic parenchyma zone, using a sliding window algorithm, which assumed the traversal of the image with a window of 56 × 56 pixels in size. If the window was situated inside the delimiting polygon and its intersection with the non-HCC regions was smaller than 0.1%, the corresponding patch was assigned to the HCC class. If the window was situated outside the polygon and its intersection with the HCC zone was smaller than 0.1%, the current patch was integrated in the cirrhotic parenchyma class. Eloquent examples of patches from each dataset are illustrated in Table 1.

For performing a reliable computerized analysis, a patch size of around 50 × 50 pixels was chosen for being able to almost integrate in the tumor region entirely and to comprise a significant number of pixels. The initially generated patches were augmented through geometrical transforms (rotation, horizontal and vertical translation, scaling and horizontal flip). Finally, 6910 HCC patches, 7148 cirrhotic parenchyma patches resulting from the GE7 dataset and 10,000 patches/class from the GE9 dataset were obtained. The classes were almost equally distributed in both datasets.

### 2.2. The Proposed Solution

**Figure 2:**
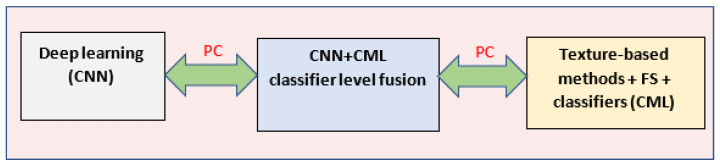
The graphical representation of the proposed methodology: the fusion between the CNN and CML methods (in the **middle**), as well as the performance comparison (PC) with the deep learning methods (**left**) and the CML methods (**right**), respectively.

In our current study, the main objective was that of enhancing the HCC automatic recognition performance through the fusion between deep learning and CML methods at the classifier level, the newly obtained performance being compared with that achieved when employing only deep learning methods and CML methods, respectively, on the experimental datasets. This methodology is illustrated in Figure 2.

#### 2.2.1. Deep Learning Techniques Involved in Our Solution

CNNs constitute deep feed-forward ANNs adequate for image recognition. Their structure was inspired from biology, the organization of the connections between the neurons resembling that of the animal visual cortex [30]. With the appearance of powerful parallel computing devices such as graphics processing units (GPU), CNNs have started to be widely used, their value being emphasized in computer vision in 2012, in the context of the ImageNet competition. The main structural elements of CNNs are the convolutional layers that are employed for compressing the input data into recognized patterns to reduce the data size and to focus on the relevant patterns [31], respectively. As presented in [32], the main power of a CNN is achieved through its deep architecture that allows to extract discriminating features at multiple abstraction levels. As for the deep learning techniques involved in our solution, we assessed both relevant and newly developed CNN-based methods, considering both classical CNN architectures, as well as transformer-based methods. Thus, we experimented with several standard architectures of the ResNet [33], InceptionV3 [34], DenseNet [35], EfficientNet_b0 [36] and ResNext [37] type, the best performance being achieved for ResNet101 and InceptionV3, respectively, for the recently developed EfficienNet_b0. Thus, the residual connections of the ResNet architecture, the inception modules of InceptionV3 and also the scaling properties of EfficientNet_b0 led to the best results in the case of the current dataset. Regarding the transformer-based methods, the best performance was achieved for ConvNext_base, while other transformers such as the Vision Transformer (ViT) and ConvNext_small [38] were also assessed. Some of these architectures were enhanced for optimizing their performances. Thus, an improved version of EfficientNet_b0, denoted EfficientNet_ASPP, was designed by introducing, before the fully connected layer, an AtrousSpatial Pyramid Pooling (ASPP) module [30], in order to extract multiscale features, and a dropout layer was also added thereafter for avoiding the overfitting phenomenon. The ASPP module, which was inserted after the usual convolutional part of EfficientNet_b0, immediately before the fully connected layers, simultaneously performed a 1 × 1 convolution and two atrous convolutions of size 3 × 3 with the rates 2 and 3, respectively. At the end, a *depthcat* layer and a global average pooling layer were added, respectively. Regarding the dropout layer, an output probability of 0.5 was associated to it. A systematic description of all these CNN architectures is provided within Table 2. It also includes the size of the deep learning feature vector, as well the name of the layer at the end of which these features were extracted.

#### 2.2.2. Conventional Techniques Involved in Our Solution

- Texture analysis methods
  Texture is an intuitive concept, inspired by the human perception, referring to the visual appearance of surfaces, particularly to the aspect of human body tissues represented within medical images. Texture can be characterized through statistical parameters, able to reveal subtle aspects upon the analyzed surface or tissue, overpassing human perception. Concerning the texture-based methods involved in our research as part of the CML approach, we analyzed both representative classical techniques, as well as more advanced techniques, developed by the authors. As classical textural features, we took into account first-order gray-level statistics, such as the corresponding arithmetic mean, maximum and minimum values, as well as second-order gray-level features, such as the Haralick parameters derived from GLCM [39], computed as described in [4]. In this group of features, we included homogeneity, energy, entropy, correlation, contrast and variance, which provided valuable information on the properties of the tissue referring to the echogenicity, heterogeneity, granularity and structural complexity. The autocorrelation index [39] was also considered, providing information on the granularity of the tissue. The Hurst fractal index was included as well in our feature set, providing information on the roughness and structural complexity of the tissue. Edge-based statistics, such as edge frequency and edge contrast [39], were also found useful in order to emphasize the structural complexity. The statistics of textural microstructures, which resulted after applying the laws convolution filters [40], were also involved in our research for the same reason. Features such as the frequency and density (arithmetic mean) of microstructures such as levels, edges, spots, waves and ripples were estimated as potentially relevant for the detection of the malignant tumors. Simultaneously, multiresolution features, in the form of the Shannon entropy computed after applying the Wavelet transform recursively twice [4], were considered able to derive subtle information on the malignant tissues, facilitating their differentiation from other tissue types. The Local Binary Pattern (LBP) also represents a powerful texture analysis method, invariant to illumination changes, particularly to those repetitive background changes due to wind or to water waves. It was firstly introduced in [41]. For obtaining these features, around each pixel, a circle of radius *R* can be considered. On this circle, *N* neighbors can be selected. For effectively achieving the LBP code, the difference between the central pixel and each of the *N* neighbors is computed. For each neighbor, if this difference is larger than 0, a code with the value of 1 is considered, otherwise, a 0 valued code is stored. The corresponding N codes constitute a number representing the LBP code. In our work, the LBP features were derived by varying the values of the *R* and *N* parameters. The following (*R*, *N*) value pairs were considered: (1,8), (2,16) and (3,24), respectively. Compressed LBP histograms with a smaller number of bins (100) were computed on each Region of Interest (ROI) of the dataset.
  We also employed advanced, original textural features elaborated by the authors, such as the edge orientation variability [4] and GCM of superior order, respectively. The superior-order GCM were defined as described in (1). According to this mathematical formula, each element of this matrix was equal with the number of n-tuples of pixels, having the values $\left(a_{1},a_{2},\cdots ,a_{n}\right)$ for the considered attribute *A*, which can stand for the intensity level, edge orientation, etc. These pixels are in a specific spatial report, defined by the displacement vectors.
  (1) $$\begin{array}{c} C_{D}\left(a_{1},a_{2},\ldots ,a_{n}\right)=\#\{(\left(x_{1},y_{1}\right),\left(x_{2},y_{2}\right),\ldots ,\left(x_{n},y_{n}\right):\\ A\left(x_{1},y_{1}\right)=a_{1},A\left(x_{2},y_{2}\right)=a_{2},\ldots ,A\left(x_{n},y_{n}\right)=a_{n},\\ |x_{2}-x_{1}\left|=\right|\vec{dx_{1}}\left|,\right|x_{3}-x_{1}\left|=\right|\vec{dx_{2}}\left|,\ldots ,\right|x_{n}-x_{1}\left|=\right|\vec{dx_{n-1}}|,\\ |y_{2}-y_{1}\left|=\right|\vec{dy_{1}}\left|,\right|y_{3}-y_{1}\left|=\right|\vec{dy_{2}}\left|,\ldots ,\right|y_{n}-y_{1}\left|=\right|\vec{dy_{n-1}}|,\\ sgn\left(\left(x_{2}-x_{1}\right)\left(y_{2}-y_{1}\right)\right)=sgn\left(\vec{dx_{1}}\cdot \vec{dy_{1}}\right),\ldots ,\\ sgn\left(\left(x_{n}-x_{1}\right)\left(y_{n}-y_{1}\right)\right)=sgn\left(\vec{dx_{n-1}}\cdot \vec{dy_{n-1}}\right))\} \end{array}$$
  The displacement vectors are defined by (2):
  (2) $$\vec{d}=\left(\left(\vec{dx_{1}},\vec{dy_{1}}\right),\left(\vec{dx_{2}},\vec{dy_{2}}\right),\ldots ,\left(\vec{dx_{n-1}},\vec{dy_{n-1}}\right)\right)$$
  In the current study, we included the Haralick features derived from the third-order GLCM. Regarding the spatial relation between the three considered pixels, they were either collinear, with the current pixel in the central position, or they formed a right-angle triangle, with the current pixel in the position of the $90^{\circ }$ angle [4]. For each configuration, the third-order GLCM was computed, the Haralick feature values being provided separately for each direction combination. A newly defined form of Textural Microstructure Co-occurrence Matrix (TMCM) was employed for the first time in the current work, assuming to compute the co-occurrence matrix after applying the k-means algorithm [40] on the ROI. In this case, the considered attributes *A* were the cluster labels assigned to each pixel as the result of the grouping algorithm. The Haralick features yielded by the second- and third-order TMCM were derived thereafter, considering several values of *k* (250 and 500), which led to significant results. The Haralick features for both second-order and third-order TMCM were computed in the same manner as for the second- and third-order GLCM. A systematic description of the texture analysis methods involved in the current research, which highlights their classical or original character, is provided in Table 3.
- Dimensionality reduction techniques
  After computing these potentially relevant textural features, the resulting vector was combined with the deep learning feature vector using specific fusion schemes described in the next subsection. These schemes involved dimensionality reduction methods from both classes of feature selection and feature extraction (KPCA). As feature selection methods, we employed both classical and bio-inspired techniques.
  Regarding the classical techniques, we employed Correlation-based Feature Subset (CFS) and Information Gain Attribute Selection (IGA) [42] that provided the best results in our previous research [5]. CFS represents a powerful method from the class of filters [42]. In the center of the corresponding algorithm, an appropriate heuristic is considered, which confers, to a certain attribute subset, a score that increases according to the strength of the correlation of this attribute with the class where the instance belongs and decreases when the same attribute is correlated with the other attributes, respectively. This method is employed together with an appropriate search algorithm (best first and genetic search) that provides all the potentially relevant attribute subsets [43]. Another representative method from the filters category is IGA. This technique assigns a score to each attribute reflecting the *Information Gain*, then it ranks the attributes in descending order based on this score. The method determines the entropy of the class *C*, before and after observing the attribute *A* [42]. The gain corresponding to the attribute *A* is given by the measure in which the attribute *A* conducts the decrease in the entropy of the class *C*. Thus, the score assigned to each attribute is computed as the difference between the entropy of the class *C* and the entropy of the class *C* obtained after observing the attribute *A*, respectively. The above-presented feature selection techniques were exploited in a combined manner by employing the intersection between the resulted feature subsets.
  From the class of the bio-inspired feature selection methods, the Particle Swarm Optimization (PSO) algorithm was considered. The elements of the particle swarm are associated to the items of the search space, these particles continuously changing their position in order to reach the optimal solution according to a well-defined criterion materialized through a fitness function [44]. In the context of the feature selection process, PSO is usually employed together with wrapper methods in order to search for the best feature subset that maximizes the performance of a given classifier. The fitness function to be minimized usually refers to the classifier error rate (i.e., 1−accuracy). In the current work, our newly defined fitness function had the form provided by (3). The current fitness function represents the weighted mean between the classification error, the ratio between the number of the selected features and the total number of features, respectively, the classification error having a higher weight associated with it (0.8). The classification error was computed as the arithmetic mean between the errors of two basic classifiers, k-nn and the Bayesian classifier.
  (3) $$f=0.8\cdot error+0.2\cdot \frac{no\_selected\_features}{no\_features}$$
  The feature extraction methods project the original feature vectors onto a new feature space, having a lower dimensionality, simultaneously highlighting important characteristics of the data. A very popular feature extraction technique, the *Principal Component Analysis (PCA)*, performs the mapping of the initial data to a lower dimensional space, where the main variation modes are emphasized. *Kernel PCA (KPCA)*, the generalization of PCA, implies the transposition of PCA in a space of larger dimensionality, built by employing the kernel function *K* of the form K=gram(X,X,kerneltype), where *kernetype* can be linear Gaussian of polynomial [45]. In our work, all the three versions of KPCA, linear, polynomial and Gaussian, respectively, were assessed. The fused vector was provided at the entrances of a powerful conventional classifier. The conventional supervised classifiers or metaclassifiers adopted in this situation were the Support Vector Machines (SVM), Random Forest (RF) and AdaBoost combined with the C4.5 algorithm for Decision Trees, respectively. These techniques, acknowledged in the domain for their increased performance, provided the best results in our former studies [5], as well as in our current study.

#### 2.2.3. Combining the Traditional and Deep Learning Techniques at the Classifier Level

**Figure 3:**
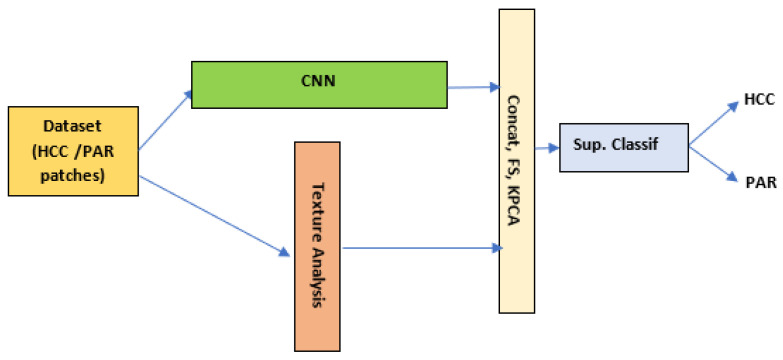
The graphical representation of the methodology for classifier-level fusion: (1) the images in the dataset are simultaneously provided to the CNN and to the texture analysis module; (2) then, the two resulting feature vectors are concatenated, feature selection methods or KPCA being eventually applied before or after the concatenation procedure; and (3) the fused feature vector is then provided at the entrance of a powerful conventional supervised classifier.

The combination (fusion) of the CNN-based methods and of the CML methods at the *classifier level* was assumed to provide the initial dataset consisting of HCC and PAR patches, at the input of a CNN classifier and to the texture analysis methods, respecitvely, as illustrated in Figure 3. Then, the deep learning features, extracted at the end of the convolutional part of the CNN, were fused with the textural feature vector through a simple concatenation or through a combination procedure that involved dimensionality reduction, such as Feature Selection (FS) or KPCA. At the end, a supervised traditional classifier was employed for completing the resulted hybrid classifier architecture to assess the classification performance. In this study, the above-described textural features formed the conventional feature vector. As for the deep learning features, they were gathered at the end of the last layer, which preceded the fully connected layers, as described within Section 2.2.1. In this context, appropriate fusion methods for yielding combined deep learning and textural feature vectors were elaborated by employing the following combination schemes: (1) the simple concatenation of the deep learning and textural feature vectors (*Concat*); (2) the concatenation of the deep learning and textural feature vectors, after the application of the classical feature selection procedure (*FS+Concat);* (3) the concatenation of the two feature vectors, followed by the application of the classical feature selection procedure (*Concat+FS*); (4) the concatenation of the two feature vectors, followed by the application of the PSO-based feature selection procedure (*Concat+PSO*); (5) the concatenation of the deep learning and textural feature vectors, after the application of the KPCA method, in order to yield the generalized principal components for each category, which were fused thereafter (*KPCA+Concat*); and (6) the concatenation of the two feature vectors, followed by the application of KPCA (*Concat+KPCA*).

Concerning the classical feature selection methods, the CFS and IGA techniques were adopted, the PSO algorithm being implemented as described in Section 2.2.2. As for KPCA, in the case of the *Concat+KPCA* fusion scheme, 500 components were extracted, while in the case of the *KPCA+concat* fusion scheme, 300 components were derived from the deep learning, as well as from the textural feature vector, in order to balance the lengths of the final feature vectors that resulted in each case. Thereafter, the correlations between the deep learning features and the textural features were analyzed in order to explain the significance of the deep learning features with respect to the visual and physical properties of the malignant tissue. For this purpose, the Pearson correlation method was employed [40].

#### 2.2.4. The Newly Defined Imagistic Model of HCC

In the context of our former research, we defined the imagistic textural model of HCC, consisting of (1) the complete set of relevant textural features which best differentiated among HCC and the visually similar classes: cirrhotic parenchyma on which HCC had evolved and benign liver tumors, respectively, as well as (2) the specific values associated with the relevant textural features: arithmetic mean, standard deviation and probability distribution [4]. In this study, this model was extended by adding the most relevant deep learning features extracted at the end from various levels of the CNNs, together with their specific values. Thus, the new set of best discriminative features (RelF) resulted by employing the most appropriate combination schemes (Comb) upon the textural (CML) and deep learning (DL) features, as illustrated in (4).

(4) RelF=Comb(DL_features,CML_features)

The newly resulting imagistic model of HCC consisted of the specific values associated to each feature of the *RelF* set and of the properties associated to the relevant feature map image, respectively, such as the arithmetic mean of the gray levels and the standard deviation, as depicted in (5). The feature map image resulted by transposing the final relevant feature vector into a gray-scale image. The discovered correlations between the textural and the deep learning features were also part of this model.

(5) $$IM=\underset{rf\in RF}{⋃}\left(mean(rf),stdev(rf),prob\_distrib(rf)\right)⋃Prop\left(feature\_map\_img\right)$$

#### 2.2.5. Performance Assessment

For classification performance assessment, the following metrics, appropriate for automatic diagnosis in the medical domain, were approached: accuracy or recognition rate, sensitivity or True-Positive (TP) Rate, specificity or True-Negative (TN) Rate and Area under ROC (AuC), respectively [40]. In our experimental context, HCC was considered the positive class, while PAR was considered the negative class. As for the automatic cancer diagnosis, both sensitivity and specificity are important, referring to the probability of the presence and lack of disease, respectively. Thus, the presence of cancer should be detected as early as possible, but the situation of erroneous cancer detection should be avoided, as those patients who are not affected by malignancy should not be sent to specific, often harmful treatments.

### 2.3. Experimental Settings

The above-mentioned techniques were implemented as follows:

- Most of the CNNs were implemented in the Matlab R2021b environment, except ConvNext_base, which was available only in Python [37].
- The conventional classifiers and the classical feature selection methods were employed with the aid of the Weka 3.8. library [43].
- KPCA for feature extraction and PSO for feature selection were implemented in Matlab R2021b.
- Most of the texture analysis methods were implemented in Visual C++, except LBP, which was implemented in Python.

Thus, the majority of the CNNs, i.e., *ResNet101*, *InceptionV3*, *EfficientNet_b0* and the improved *EfficientNet_b0* were implemented in Matlab R2021b, with the aid of the Deep Learning Toolbox [46]. The improved *EfficientNet_b0* architecture, enhanced with an ASPP module and a dropout layer, was built in the *Deep Network Designer* environment, starting from the *EfficientNet_b0* architecture, as described in Section 2. All these networks were trained in the following conditions:

- The Stochastic Gradient Descent with Momentum (SGDM) strategy was employed;
- The learning rate was set to 0.0002;
- The momentum was set to 0.9;
- The minibatch size was set to 30;
- The duration of the training process was 100 epochs.

These hyperparameter values were set for achieving an accurate, efficient learning process and to simultaneously avoid overtraining, as well as considering the memory constraints of the computer (the minibatch size). All the above-mentioned networks were pretrained on the ImageNet dataset, the training being refined thereafter using the specific data from the B-mode ultrasound images of our datasets. The ConvNext-type CNN, as a recent, powerful architecture, was implemented in Python with the aid of the *Torchvision* library [37]. It was trained in a similar manner, using the same strategy and the same values of the hyperparameters as those adopted for the other CNN architectures. The last layer was reshaped for all the considered networks in order to provide only two outputs, which corresponded to the HCC and PAR classes. The feature maps were derived from the trained CNNs, as mentioned within Section 2.2.1, using specific Matlab and Python functions (*activations* and *get_activations*, respectively). Regarding the dimensionality reduction techniques, the method of KPCA was employed in Matlab 2021, with the aid of the Matlab-Kernel-PCA toolbox [47], the linear, third-degree polynomial and Gaussian kernels being experimented on. The PSO-based feature selection method was implemented in Matlab as well, using a specific framework [48]. The classical feature selection methods were implemented by using the Weka 3.8. library [43]. Thus, the CfsSubsetEval(CFS) technique was implemented with BestFirst search, while the InfoGainAttributeEval method was employed in conjunction with Ranker search. The conventional classifiers were employed, as well, using the Weka 3.8. library [43], as follows:

- The John Platt’s Sequential Minimal Optimization (SMO) algorithm [43], the Weka equivalent of SVM, was assessed, the best performance resulting for the polynomial kernel of 3rd degree.
- The AdaBoost metaclassifier was assessed for 100 iterations in conjunction with the J48 method, the equivalent of the C4.5 algorithm in Weka.
- The RandomForest (RF) technique of Weka was adopted as well.

Some of the textural features were computed using our own Visual C++ software modules, as described in Section 2.3, independently on orientation, illumination and scale, after applying a median filter for speckle noise reduction. The LBP features were computed in Python using the *Numpy* library.

All these experiments were conducted on a computer having an i7 processor of 2.60 GHz, 8 GB of internal (RAM) memory and an Nvidia Geforce GTX 1650 Ti GPU. Regarding *the performance evaluation strategy* for the CNN-based methods, 75% of the data constituted the training set, 8% of the data stood for the validation set and 17% of the data were integrated in the test set. For the conventional classifiers, 75% of the data constituted the training set, while 25% of the data were integrated in the test set.

## 3. Results

### 3.1. CNN Performance Assessment

In Table 4, the values of the classification performance parameters for the individual CNNs, obtained through transfer learning, on both considered datasets were provided. The maximum values resulted for each classification performance parameter, for each dataset, were highlighted with *bold*. Thus, for the first dataset (GE7), the highest classification accuracy, the highest sensitivity, the most increased specificity and the best AUC resulted for the ResNet101 architecture. EfficientNet_ASPP, the improved version of the EfficientNet_b0 architecture, led to an increase in the classification performance in comparison with EfficientNet_b0 regarding all the assessed metrics.

For the second dataset, GE9, InceptionV3 provided the best classification accuracy, followed by ResNet101. The best sensitivity resulted for ResNet101, while the highest specificity was achieved for ConvNext_base. The most increased AuC was obtained for InceptionV3. For the GE9 dataset, EfficientNet_ASPP, the enhanced version of EfficientNet, led, once again, to an increased classification performance in terms of accuracy, sensitivity and AUC, in comparison with the original, EfficientNet_b0 architecture. As we can notice, the values of the classification performance parameters achieved for the first dataset, GE7, were higher than the values resulted for the same parameters in the case of the GE9 dataset. The reason could be the fact that the GE7 dataset included a smaller number of HCC patches that were manually selected, emphasizing a specific HCC region that in many cases was visually different from the cirrhotic parenchyma, while in the case of the GE9 dataset, the patches were automatically selected from the entire tumor surface.

### 3.2. Assessing the Performance of the Textural Features through Conventional Classifiers

In Table 5, the values of the classification performance parameters obtained on each dataset by providing the relevant textural features at the entrances of conventional classifiers are depicted. The maximum values are highlighted in *bold* for each parameter for each dataset. For the first dataset, GE7, the highest classification accuracy and the best sensitivity, as well as the best AUC, resulted for AdaBoost, while the highest specificity was obtained for SVM. As for the GE9 dataset, the most increased accuracy, the best sensitivity and the best specificity, as well as the highest AUC, resulted for AdaBoost. As we can infer by comparing Table 4 and Table 5, the values of the classification performance parameters achieved through the CNN techniques were comparable to those obtained when employing conventional CML for both datasets. However, the maximum values were achieved for the CNNs in most of the situations.

### 3.3. Assessing the Performance of the Combination between the Textural and CNN Features

#### 3.3.1. Performance Assessment on the GE7 Dataset

Within Table 6, the arithmetic mean of the values of the performance parameters obtained on the GE7 dataset trough the three considered conventional classifiers for each combination of the textural features with deep learning features derived from a certain type of CNN are depicted. For each parameter, the highest values are emphasized in bold in the case of each CNN. As we can notice, the absolute maximum of the mean accuracy, 97.47%, as well as the absolute maximum of the mean sensitivity, 97.53%, resulted when combining ResNet101 with the textural features through the *Concat+FS* fusion scheme; the absolute maximum of the average specificity, 98.63%, resulted when combining InceptionV3 with the textural features for the *KPCA+concat* fusion scheme, while the absolute maximum of mean AUC, 97.86%, resulted when combining ResNet101 with the textural features through the PSO scheme. The best overall accuracy of 98.23% resulted when the InceptionV3 CNN architecture was involved for the *KPCA+concat* combination scheme. In the case of AdaBoost, the best overall sensitivity of 98.2% resulted when ResNet101 was involved; in the case of the *KPCA+concat*, for the AdaBoost metaclassifier, the highest overall specificity of 98.9% was achieved for the *KPCA+concat* fusion scheme in the case of the RF classifier when the InceptionV3 CNN was involved, while the highest AUC of 99.3% resulted for *KPCA+concat*, in the case of the RF classification technique, when the EfficientNet_ASPP CNN architecture was employed.

**Figure 4:**
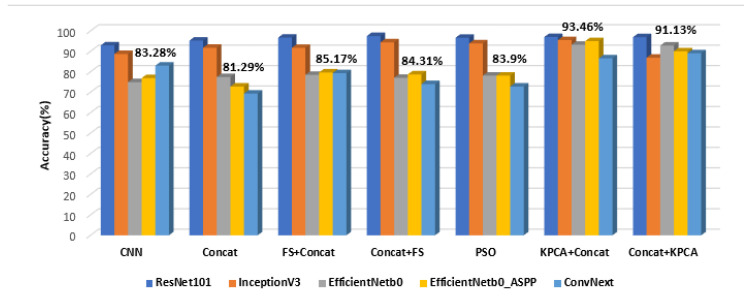
The comparisons between the average accuracy values obtained for each combination scheme for the considered CNN architectures in the case of the GE7 dataset.

**Figure A1:**
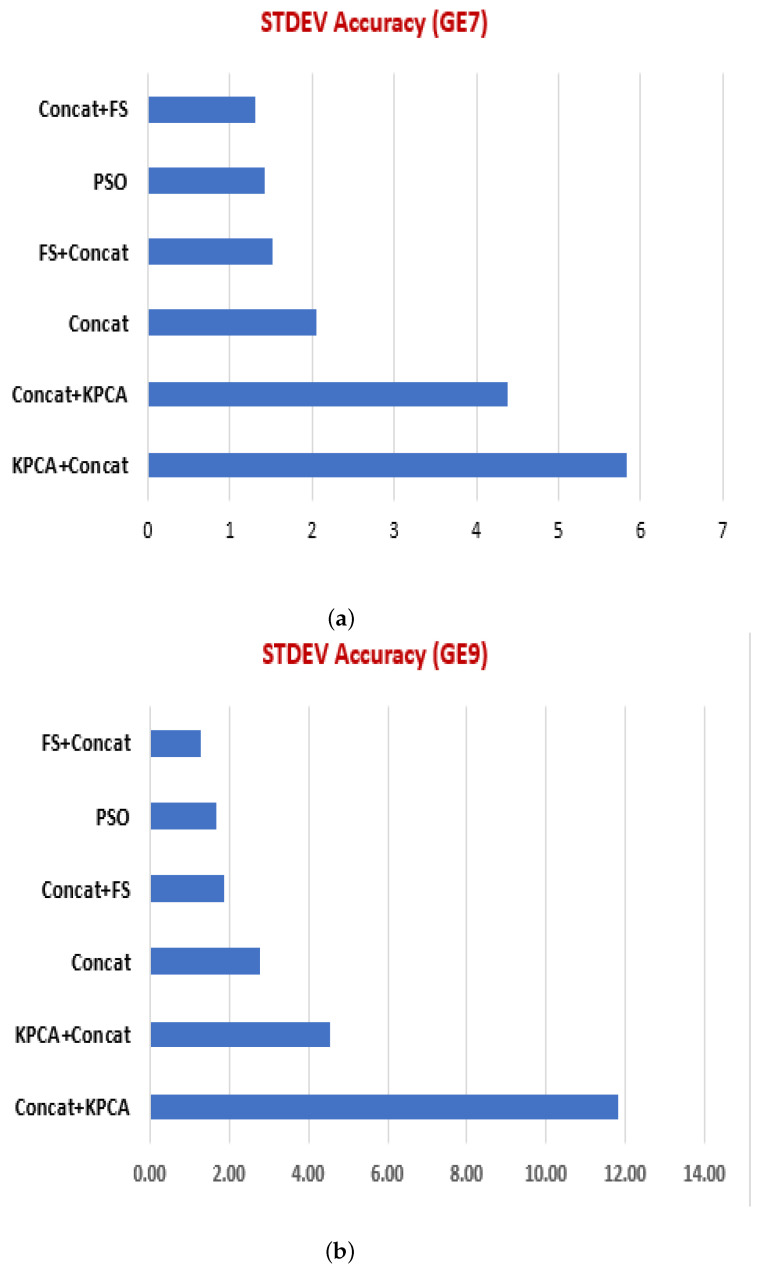
The standard deviations of the accuracy values for each combination scheme: (**a**) for the GE7 dataset; (**b**) for the GE9 dataset.

In Figure 4, the comparisons between the average accuracy values corresponding to the considered combination schemes, in the case of each CNN, are illustrated. These values are also compared with the accuracy values obtained when using only the CNN by itself. Above each group, which corresponds to a certain combination scheme, the arithmetic mean of the accuracy values per group was depicted. As it can be noticed, the performance of the considered combination schemes overpassed that of the individual CNNs in most of the situations. Moreover, all the combination schemes involving feature selection and KPCA provided a better performance than that achieved when employing a simple concatenation between the CNN and the textural feature vectors. Thus, a maximum average accuracy of 93.46% was achieved in the case of *KPCA+Concat*, followed by an average accuracy of 91.13% achieved for the *Concat+KPCA* combination. Regarding the CNN architectures, ResNet101, followed by InceptionV3, provided the highest accuracy values for most of the considered fusion schemes. In Appendix A, within Figure A1, the standard deviations of the classification accuracy values for each combination scheme are provided. As it can be noticed, in the case of the GE7 dataset, the smallest standard deviation of 1.3 was achieved for the *Concat+FS* combination scheme and then by the PSO combination scheme (1.43), followed by the *FS+Concat* fusion scheme (1.51). On the last position, the *KPCA+concat* fusion scheme was situated, the standard deviation being 5.83.

#### 3.3.2. Performance Assessment on the GE9 Dataset

Table 7 illustrates the arithmetic mean of the performance parameters resulted from the GE9 dataset for each combination of the textural features, with deep learning features extracted from a certain CNN, for each fusion scheme. For each parameter, for each type of CNN, the highest values were emphasized in bold. We can infer that the maximum overall value of the mean accuracy of 98.01%, the maximum mean sensitivity of 98.26%, the maximum mean specificity of 97.9% and the maximum mean AUC of 94.16% were obtained for the *KPCA+concat* fusion scheme when ResNet101 was involved. As for the individual values, obtained through each conventional classifier, the best overall accuracy of 98.9% and the best overall specificity of 98.6% resulted for the combination between InceptionV3 and the textural features for the *KPCA+concat* combination scheme when employing the AdaBoost metaclassifier. The best sensitivity of 99.2% was achieved in the case of *KPCA+concat* for AdaBoost for the combination between ResNet101 and the textural features, while the most increased AUC of 99.7% resulted for *KPCA+concat* for the RF classification technique in the case when InceptionV3 was combined with the textural features.

**Figure 5:**
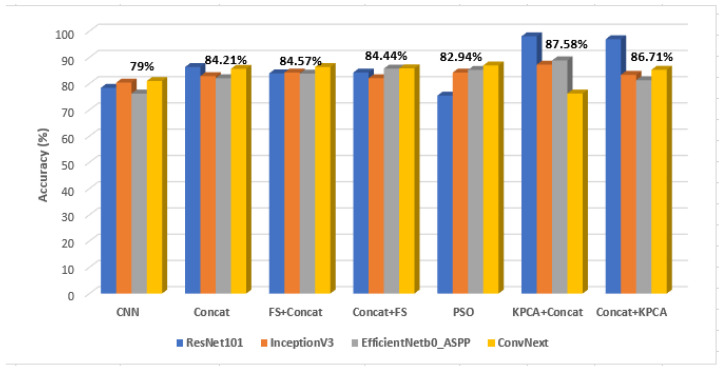
The comparisons between the average accuracy values obtained for each combination scheme for the considered CNN architectures in the case of the GE9 dataset.

Within Figure 5, the comparison among the arithmetic mean of the accuracy values for each combination scheme for the considered CNN architectures is depicted, the arithmetic mean of the accuracy values per fusion scheme being illustrated above each corresponding group. The best average accuracy of 87.58% was achieved in the case of KPCA, followed by concatenation, while the second best mean value of 86.71% was obtained in the case of concatenation followed by KPCA. The information inferred by Figure 5 confirms that provided by Figure 4, the ranking of the fusion schemes being almost similar according to these figures. It must also be noticed that simply performing concatenation led to worse results than all the other combination schemes. Concerning the best performing CNN architectures, ResNet101, as well as ConvNext_base, provided very good performances in most of the situations. Regarding the standard deviations of the accuracy values achieved for each fusion scheme in the case of the current dataset, according to Figure A1, the smallest standard deviation resulted from the *FS+Concat* fusion scheme (1.27), followed by PSO (1.66) and then by *Concat+FS* (1.89), *Concat+KPCA* (11.83) being situated on the last position.

### 3.4. The Newly Defined Imagistic Model of HCC

**Figure 6:**
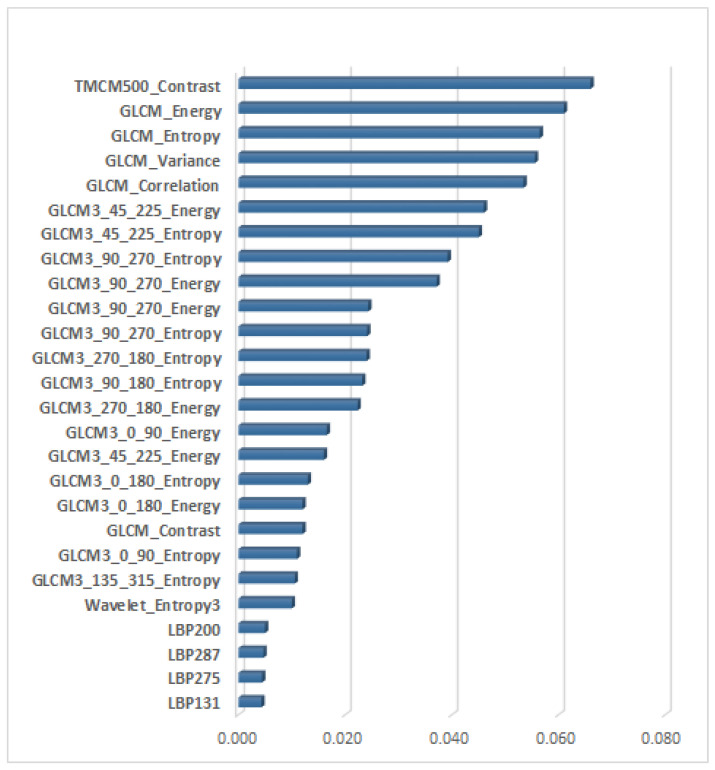
The ranking of the most relevant textural features among the combined feature vector for the GE7 dataset.

**Figure 7:**
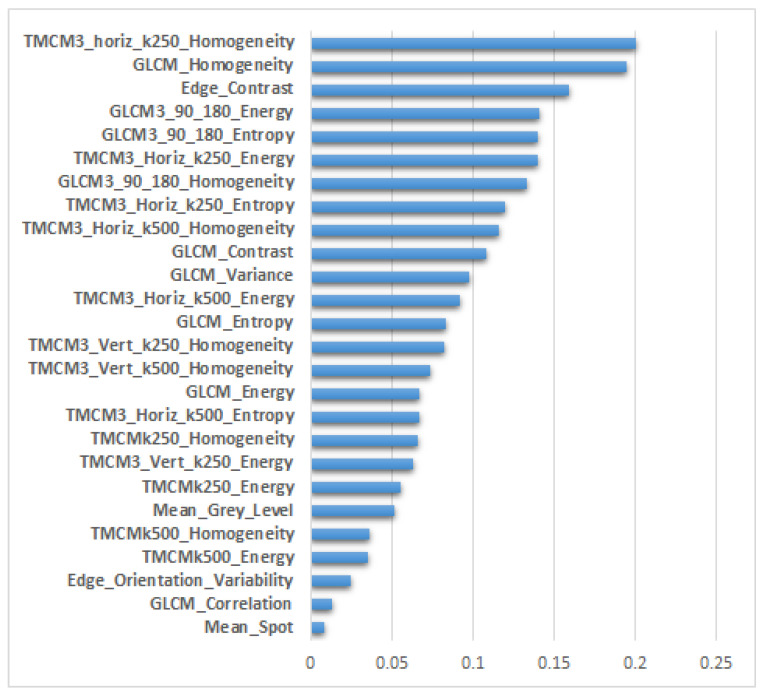
The ranking of the most relevant textural features through the combined feature vector for the GE9 dataset.

The relevance of the considered textural features in the classification process was assessed in the context of the entire feature vector when considering their combination with the CNN features. In Figure 6 and Figure 7, the ranking of the most relevant textural features from each dataset is provided, this ordering being derived after the application of the IGA technique upon the combined feature vector, containing both textural and CNN features. The length of each line of the graphic represents the arithmetic mean of the particular scores resulted from the application of IGA upon the combination between the textural features and the CNN features provided by each CNN technique. In both these figures, we notice, on the first positions, the presence of the features derived from the generalized co-occurrence matrices, including the second- and third-order TMCM and GLCM features.

For the GE7 dataset, the most relevant feature is the contrast obtained from the TMCM matrix, computed for k = 500, having an average score of 0.066, the maximum average score among the entire feature set being 0.357. The Haralick features derived from the GLCM matrices of order 2 and 3, computed for various directions of the displacement vectors, followed thereafter, emphasizing the heterogeneous, chaotic structure of the tumor tissue through the GLCM_Energy, GLCM_Entropy and GLCM_Variance. They also revealed differences in granularity between the HCC and PAR tissue classes, through the GLCM_Correlation. Towards the end of the ranking, we notice the presence of the entropy computed after the application of the Wavelet transform at the first level on the third component (high–low) and of the LBP features, respectively, emphasizing again the chaotic structure, as well as the complexity of the malignant tumor.

**Figure 8:**
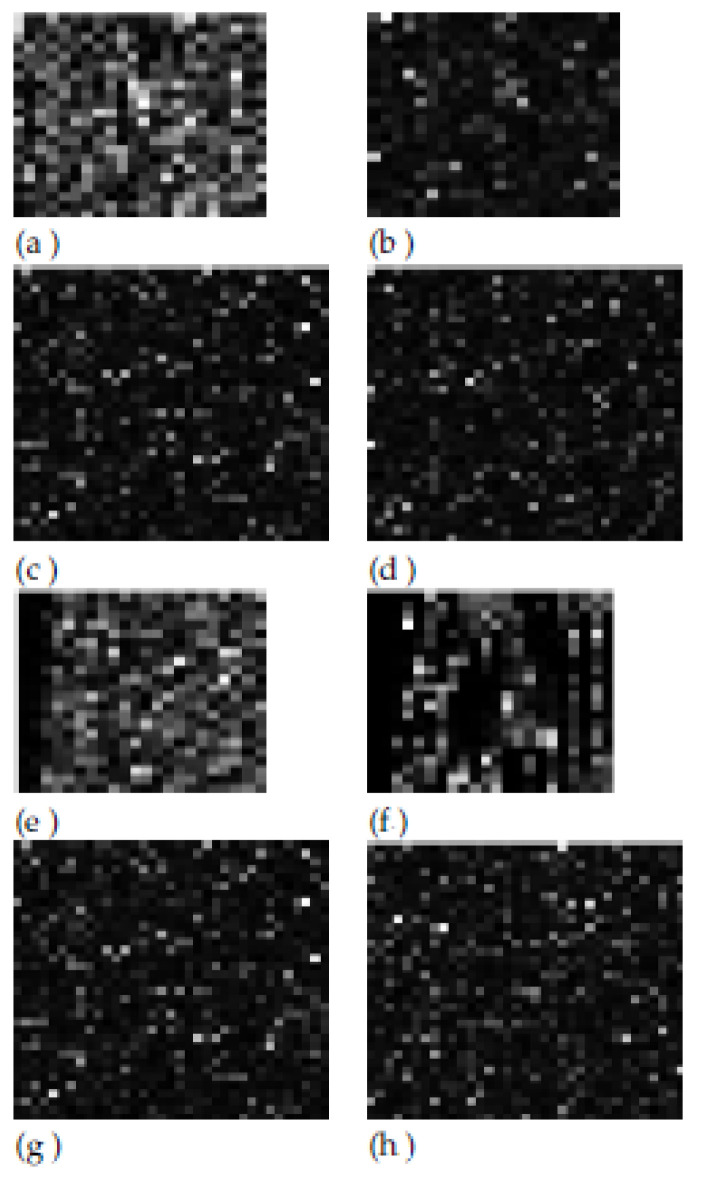
The comparisons between the activation maps for EfficientNet_ASPP for the combination between EfficientNet_ASPP and the textural features through the *Concat+KPCA* fusion scheme, respectively: (**a**,**b**,**e**,**f**) the maps for EfficientNet_ASPP combined with the textural features for HCC (**a**,**e**), respectively, PAR (**b**,**f**); (**c**,**d**,**g**,**h**) the maps achieved in the case of EfficientNet_ASPP for HCC (**c**,**g**), respectively, for PAR (**d**,**h**); (**a**–**d**) stand for GE7; (**e**–**h**) stand for GE9.

**Figure A2:**
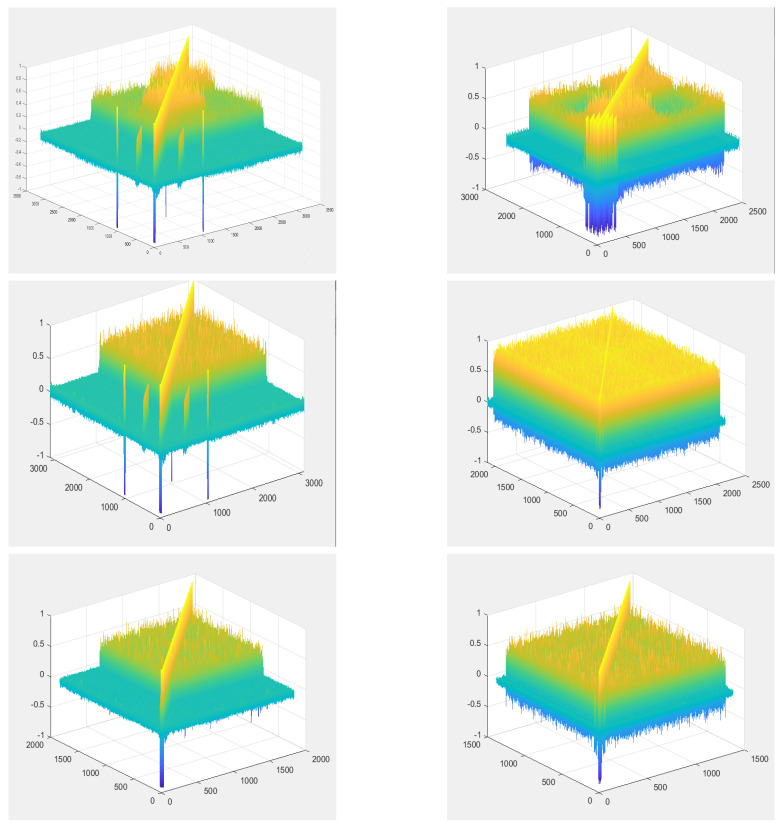

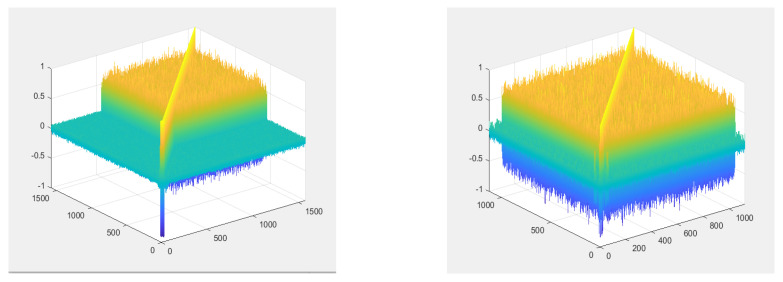
The correlations between the textural and CNN features.

As for the GE9 dataset, as depicted in Figure 7, the first position among the whole feature vector was occupied by the homogeneity derived from the third-order TMCM matrix when the displacement vectors were collinear on the horizontal direction and the value of k was 250, being associated with the highest relevance score of 0.2, immediately followed by the same parameter derived from the second-order GLCM matrix. These attributes emphasized the differences in homogeneity between HCC and the cirrhotic parenchyma on which it had evolved. Towards the end of the ranking, we notice the presence of the edge orientation variability, of the GLCM correlation, of the density of the spot microstructures computed after employing the Laws’ convolution filters, denoting both the complexity of the HCC tissue as well as the difference in granularity between the HCC and PAR tissues, respectively. The correlations between the textural features and the CNN features were evaluated as well for each CNN architecture on both datasets. The plots of the pairwise correlations between the considered textural features and the CNN features, assessed with the aid of the Pearson correlation method, are depicted in Appendix B, Figure A2. The fact that there exist increased correlations among the CNN features themselves can be noticed; there are some medium correlations among the textural features, as well as smaller correlations between the textural features and the CNN features, respectively. As for the correlations between the textural features and the CNN features, the highest correlations were those met for the GLCM_variance with three ResNet101 features for the GE9 dataset, the maximum correlation coefficients being 0.429, 0.26 and 0.17, respectively, followed by the correlations met between the TMCM500_contrast and the InceptionV3 features on the GE7 dataset of 0.197, 0.194, 0.184, 0.176 and 0.171, then by the correlations obtained on the GE7 dataset between the GLCM3_45_225_energy, the GLCM_90_270_energy with the EfficientNet_ASPP features of 0.179, by those between the TMCM500_contrast and the ResNet101 features on the GE9 dataset of 0.176, respectively, and by those between the GLCM_homogeneity and the InceptionV3 features on the GE7 dataset of 0.124. As part of the newly approached textural model, the comparisons between the activation maps corresponding to the CNN features derived from EfficientNet_ASPP and those obtained from the fusion of these types of CNN features with the textural features when employing the *Concat+KPCA* combination scheme, for both datasets, GE7 and GE9, are depicted in Figure 8. The *Concat+KPCA* combination scheme was taken into account as being one of the best performing fusion schemes that also transformed the elements of the original concatenated vector, yielding more refined fused features, which emphasized the main variation modes. These activation maps were achieved by adequately reshaping the feature vectors for obtaining a maximal square image. The first and second lines correspond to the results achieved on the first dataset, GE7, while the third and fourth lines correspond to the results obtained on the GE9 dataset. The left-hand-side column corresponds to the HCC class, while the right-hand-side column stands for the PAR class. The first and third lines correspond to the activation maps achieved in the case of the fusion between the EfficientNet_ASPP CNN features and the textural features, while the second and fourth lines to the activation maps obtained in the cases when only the EfficientNet_ASPP CNN features were taken into account. It can be noticed that, in the case of HCC, the patterns are more heterogeneous and the frequency of the increased pixel values is larger than in the case of PAR, these differences being more emphasized for the activation maps corresponding to the fusion between the CNN and textural features. This remark is confirmed by the numerical differences obtained between the corresponding values of the mean gray levels and standard deviations of these maps, respectively. Thus, in the case of the EfficientNet_ASPP activation maps obtained for the GE7 dataset, the difference between the standard deviations for the HCC and PAR classes was 0.0034, while the difference between the HCC and PAR gray-level means was 0.0091. For the same dataset, when considering the activation maps for the fusion between the CNN and textural features, the difference between the standard deviations corresponding to the HCC and PAR classes was 0.0169, while the difference between the gray-level means was 0.2545. In the case of the EfficientNet_ASPP activation maps achieved for the GE9 dataset, the difference between the standard deviations for the HCC and PAR classes was 0.0042, while the difference between the HCC and PAR gray-level means was 0.0079. When taking into account the activation maps for the combination between the CNN and textural features, the difference between the HCC and PAR standard deviations was 0.0129, the difference between the gray level means being 0.0759.

## 4. Discussion

**Figure 9:**
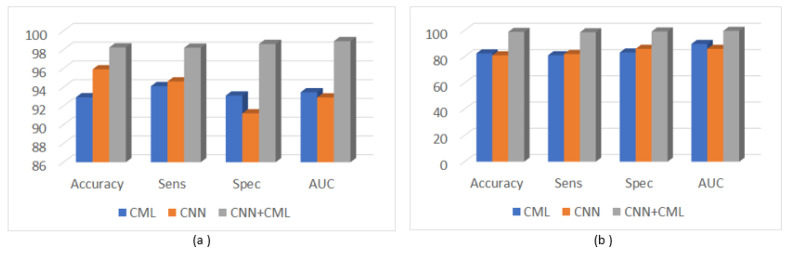
Comparison between the maximum value of the classification performance metrics for the three considered classes of methods: CML, CNN and the combination between CML and CNN (CML+CNN): (**a**) on the GE7 dataset; (**b**) on the GE9 dataset.

As it results from the previous sections, as well as from Figure 9, the combinations between the CNN-based techniques and the CML techniques at the classifier level achieved better classification performances in terms of accuracy, sensitivity, specificity and AUC, in comparison with the individual application of each class of methods.

The best classification performances were achieved for *KPCA+concat*, followed by *concat+KPCA*, *FS+concat* being situated on the third position. However, the standard deviations of the accuracy values were best when employing feature selection before or after concatenation, as it results from Figure A1 form Appendix A. Regarding the KPCA technique, the best results, in all the considered situations, were achieved when employing the Gaussian kernel. It can also be noticed that firstly applying FS and KPCA, followed by concatenation, provided better classification performances than when applying concatenation and then FS and KPCA, respectively. The conventional dimensionality reduction methods led to higher classification performances than the bio-inspired feature selection method, based on PSO, when being applied in the same conditions. Concerning the CNN architectures that were combined with the conventional techniques, the best results were provided when involving ResNet101, followed by those obtained when InceptionV3 was involved. Thus, the residual connections that contributed to overpassing the gradient vanishing problem, as well as the inception modules, significantly contributed to the enhancement of the classification performance. Convnext_base, the transformer-based architecture involved in our experiments, yielded a very good classification performance as well, especially in the case of the GE9 dataset. The newly designed EfficientNet_ASPP architecture also provided satisfying results, overpassing EfficientNet_b0 in many situations. As for the conventional classifiers, as it results from the previously presented experiments, the best classification performance was achieved by AdaBoost combined with decision trees, followed by RF and SVM, respectively. For the last-mentioned classifier, the best performance resulted when considering the 3rd-degree polynomial kernel. The computational efficiency of our solution is also satisfying, as most of the CNN architectures and most of the conventional classifiers that led to the best solution were based on less complex algorithms, dimensionality reduction also being performed upon the involved feature vectors. Regarding the textural features involved in the current work, they demonstrated their importance when assessed together with the deep learning features, achieving relevance values situated in most cases in the interval 0.05–0.2, slightly below the maximum relevance value, around 0.3, of the entire feature vector. The contrast and homogeneity derived from the TMCM matrix confirmed the heterogeneity, as well as the complex structure of the HCC tissue, as compared with that of the cirrhotic parenchyma on which it had evolved. These properties are due to the coexistence of multiple tissue types, as well as to the rich vascularization of HCC. Other relevant textural features, such as the correlation derived from the GCM, revealed differences in granularity between HCC and the cirrhotic parenchyma on which it had evolved. Some correlations between the textural features and the deep learning features, assessed through the Pearson Correlation Coefficient, resulted as well, especially between the second- and superior-order textural features, derived from the newly defined GCM, and the ResNet101, InceptionV3 and EfficientNet_ASPP CNN features, respectively. These correlations revealed the capacity of the deep learning features to emphasize the properties of the HCC and cirrhotic parenchyma tissues.

The comparisons with the already existing state-of-the-art results, in terms of classification performance metrics, are depicted in Table 8. For assessing the significance of the improvements for each state-of-the-art method, a specific metric was computed, expressed as an average difference according to the formula (6). In (6), Acc, Sens, Spec and AUC were the performance metrics corresponding to the current work, while $Acc_{sa}$, $Sens_{sa}$, $Spec_{sa}$ and $AUC_{sa}$ were the metric values corresponding to the state-of-the-art techniques.

(6) $$Avg\_dif=\frac{\left(Acc-Acc_{sa}\right)+\left(Sens-Sens_{sa}\right)+\left(Spec-Spec_{sa}\right)+\left(AUC-AUC_{sa}\right)}{4}$$

Thus, the maximum obtained classification performances in the current work overpassed the maximum performance achieved in the research paper [7]. In [7], the authors assessed the combinations, at the classifier level, between representative CNN architectures, such as ResNet101, InceptionV3 and EfficientNet_b0, on the GE9 dataset. In this case, the average difference in the performances was −1.002. The maximum classification performance achieved in the current work also overpassed that reported in [24], the average difference between the corresponding metrics being −14.38, which is larger than that which previously resulted. Thus, the method described in [24] was reproduced in the current work in the following manner: the DenseNet201 and ResNet50 CNN architectures were trained in Matlab2021 using the Deep-Learning toolbox, then our conventional FS methodology based on the combination between the CFS and IGA techniques was applied on each CNN feature vector. The experiments were performed on the most recently acquired dataset, GE9. Thereafter, the results were concatenated and the SMO classifier was applied with the aid of the Weka 3.8 library. The method described in [27] was also considered for comparison. In order to reproduce this method, we trained a VGG-16 CNN on the GE9 dataset, using the same training parameters as specified in this paper. Concerning the conventional feature vector, for characterizing the local shape, we also derived HOG features on the GE9 dataset, using Matlab-specific functions, which were added to the previously extracted textural features. Thereafter, the Symmetrical Uncertainty feature selection technique in conjunction with Ranker was applied in Weka 3.8 [43] in order to retain the most important features from each feature vectors, the resulting conventional and deep learning features being concatenated thereafter. As it can be noticed from Table 8, an average difference of −7.43 resulted between the corresponding classification performance parameters of this state-of-the-art method and the maximum performance our current research, respectively. Regarding the computational complexity of the methods analyzed in this paragraph, we can infer that our methodology is more efficient. Thus, the approaches presented in [7,24] required training two types of CNNs, as well as a conventional classifier, while our technique required training only one CNN and a traditional classifier for the best solution. Moreover, in the case of the technique described in [27], a VGG-type network corresponded to the best solution, this being a complex network having many parameters, so that the training time was much more increased than that required for our CNNs.

Moreover, the current work approaches a similar subject and methodology as the research described in [25]. However, in our approach, the HCC automatic recognition was performed in a noninvasive and efficient manner based on ultrasound images, while in [25], the authors conducted their analysis on MRI images, which might involve additional costs and risks. One of the similarities between these two approaches is the application of feature selection on the concatenated vector, followed by the employment of a traditional classifier. While in [25], a single, complex feature selection procedure was applied upon the combined feature vector, followed by the employment of the SVM classifier, in the current approach, multiple fusion methods, involving various dimensionality reduction methods were assessed, followed by the application of various conventional classification techniques, including SVM.

## 5. Conclusions

The fusion between CNN-based techniques and conventional ML methods based on advanced texture analysis proved to be very efficient, leading to increased classification accuracies higher than 95% in many situations. The combination schemes that provided the best results were *KPCA+Concat*, *Concat+KPCA* and *FS+Concat*, respectively, highlighting the role of the KPCA technique in this context. The computational efficiency of our solution was also satisfying, as discussed in Section 4. A new approach of the imagistic textural model of HCC was also elaborated, emphasizing the relevant textural features and the capacity of the newly resulted hybrid feature maps to differentiate between the HCC and PAR classes, as well the correlations between the deep learning features and the textural features. It resulted that many deep learning features were correlated with the textural features, the deep learning features also being able to reveal the HCC and PAR tissue properties. All the resulted relevant features confirmed the heterogeneous and complex structure of the HCC tissue, also revealing differences in granularity between the HCC and PAR tissue classes. Thus, the newly elaborated methodology can be appropriate for the computer-aided and automatic diagnosis of HCC. The corresponding classification performances overpassed those obtained when considering the CNN methods and conventional ML methods by themselves, as well as those resulted from the case of some representative state-of-the-art methods. As future developments, we aim to involve multiple medical image types, such as CT and MRI in this analysis, as well as to refer to multiple classes of tumors, such as pancreatic and renal tumors, including benign tumors as well. Concerning the fusion methods, the Canonical Correlation Analysis [49] is also targeted for classifier-level fusion, while decision-level fusion will also be considered.

## Figures and Tables

**Table 1:** Relevant examples from the two considered datasets GE7 and GE9.

Dataset	Class			
GE7	HCC	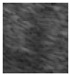	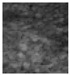	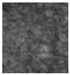
	PAR	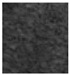	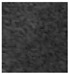	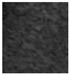
GE9	HCC	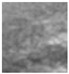	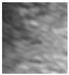	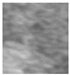
	PAR	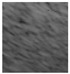	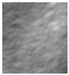	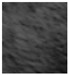

**Table 2:** The description of the CNN architectures.

CNN	Original Impr.	Last Layer	Vector Size
ResNet101	-	*pool5*	2048
InceptionV3	-	*avg_pool*	2048
EfficientNet_b0	dropout layer	*GlobAvgPool*	1283
EfficientNet_ASPP	ASPP module	*gapool*	1283
ConvNext_base	-	*adaptiveAvgPool2d*	1024

**Table 3:** The texture analysis methods involved in our research.

Texture Analysis Method	Classical/Original
2nd-order GLCM and Haralick features	Classical
Autocorrelation index	Classical
Edge frequency, Edge contrast	Classical
Density and frequency of textural microstructures (Laws)	Classical
Shannon entropy computed after the application of the Wavelet transform	Classical
LBP features	Classical
Edge orientation variability	Original
GCM (3rd-order GLCM, 2nd- and 3rd-order TMCM) and Haralick features	Original

**Table 4:** Results obtained using transfer learning.

Dataset	Method	Accuracy	Sensitivity	Specificity	AUC
	ResNet101	**95.9%**	**95.6%**	**91.2%**	**93.4%**
	InceptionV3	88.7%	88.8%	88.6%	89%
GE7	EfficientNet_b0	74.93%	72.9%	77.5%	75.2%
	EfficientNet_ASPP	76.9%	77.4%	76.1%	76.75%
	ConvNext_base	83%	78%	88%	83%
	ResNet101	78.4%	**82.0%**	75.5%	78.75%
	InceptionV3	80.39%	81.63%	79%	**86%**
GE9	EfficientNet_b0	74.32%	75.22%	73.22%	82%
	EfficientNet_ASPP	76.2%	79.8%	73.22%	76.51%
	ConvNext_base	**81%**	75%	**86%**	80.50%

**Table 5:** Results obtained on the set of relevant textural features through conventional classifiers.

Dataset	Method	Acc.	Sens.	Spec.	AUC
	SMO (poly grd.3)	92.85%	92.6%	**93.1%**	92.85%
GE7	AdaBoost+J48	**92.92%**	**94.1%**	92.8%	**93.45%**
	RF	89.9%	93.3%	88.5%	90.9%
	SMO (poly grd. 3)	78.136%	77.9%	78.4%	78.1%
GE9	AdaBoost+J48	**82.5%**	**81.1%**	**83.2%**	**89.7%**
	RF	75.85%	69.4%	82.1%	84.5%

**Table 6:** Results obtained on GE7 through various combination methods.

Combination	Fusion Method	Acc.	Sens.	Spec.	AUC
ResNet101+TF	Concat	95.25%	95.4%	95.13%	93.03%
	FS+Concat	96.71%	95.8%	97.76%	96.1%
	Concat+FS	**97.48%**	**97.53%**	**97.56%**	92.1%
	PSO	96.65%	95.26%	98%	**97.86%**
	KPCA+Concat	97.01%	97.26%	96.8%	93.16%
	Concat+KPCA	96.92%	95%	96.76%	91.53%
InceptionV3+TF	Concat	91.74%	92.43%	91.1%	95.7%
	FS+Concat	91.69%	95%	93.8%	**97.06%**
	Concat+FS	94.39%	95.2%	90.2%	94.53%
	PSO	93.87%	**96.4%**	91.33%	95.86%
	KPCA+Concat	**95.49%**	92.16%	**98.63%**	96.6%
	Concat+KPCA	86.87%	88.36%	85.4%	90.96%
EfficientNet_b0+TF	Concat	77.42%	74.06%	81.1%	81%
	FS+Concat	78.48%	77.76%	79.26%	82.78%
	Concat+FS	77.03%	71.43%	80.9%	80.66%
	PSO	78.1%	78%	78.2%	82.6%
	KPCA+Concat	**93.22%**	**90.63%**	95.26%	**94.33%**
	Concat+KPCA	92.8%	90.33%	**95.63%**	94.26%
EfficientNet_ASPP+TF	Concat	72.75%	71.13%	74.4%	78.2%
	FS+Concat	79.67%	81.06%	78.3%	84%
	Concat+FS	78.7%	79.6%	77.66%	83.23%
	PSO	78.1%	78%	78.2%	82.6%
	KPCA+Concat	**94.99%**	**92.53%**	**96.9%**	**95.96%**
	Concat+KPCA	90.01%	90.43%	91.53%	93.53%
Convnext_base+TF	Concat	69.33%	69.2%	69.43%	74.1%
	FS+Concat	79.31%	83.4%	80.7%	88.4%
	Concat+FS	73.97%	73.16%	74.8%	78.3%
	PSO	72.77%	72.23%	73.5%	76.66%
	KPCA+Concat	86.57%	86.23%	82.53%	87.16%
	Concat+KPCA	**89.03%**	**88.5%**	**90.23%**	**93.4%**

**Table 7:** Results obtained on GE9 through various combination methods.

Combination	Fusion Method	Acc.	Sens.	Spec.	AUC
ResNet101+TF	Concat	86.3%	84.6%	86.73%	90.26%
	FS+Concat	83.9%	88%	79.8%	88.96%
	Concat+FS	84.22%	87.4%	81.03%	89.1%
	PSO	75.42%	76.73%	74.16%	79.56%
	KPCA+Concat	**98.01%**	**98.26%**	**97.9%**	**94.16%**
	Concat+KPCA	96.92%	96%	97.76%	92.53%
InceptionV3+TF	Concat	82.85%	86.93%	78.66%	87.8%
	FS+Concat	84.21%	87.4%	81.03%	89.1%
	Concat+FS	82.04%	86.9%	76.73%	85%
	PSO	84.2%	87.46%	80.93%	**89.36%**
	KPCA+Concat	**87.23%**	**89.9%**	82.86%	**86.36%**
	Concat+KPCA	83.33%	82.9%	**84.16%**	88.66%
EfficientNet_ASPP+TF	Concat	82.04%	83.36%	77.56%	87%
	FS+Concat	83.83%	88.16%	79.3%	88.73%
	Concat+FS	85.72%	88.66%	80.66%	89.43%
	PSO	85.2%	88.46%	81.93%	90.36%
	KPCA+Concat	**88.86%**	**89.8%**	**87.76%**	**92.53%**
	Concat+KPCA	81.29%	87.06%	71.93%	84.33%
ConvNext_base+TF	Concat	85.63%	84.6%	86.73%	90.26%
	FS+Concat	86.33%	87.06%	**87.06%**	91.36%
	Concat+FS	85.79%	86.7%	84.93%	89.2%
	PSO	**86.94%**	87.06%	79.8%	**91.93%**
	KPCA+Concat	76.22%	76.63%	75.53%	78.16%
	Concat+KPCA	85.48%	**88.76%**	81.86%	90.16%

**Table 8:** Comparisons with other relevant state-of-the-art approaches.

Method	Accuracy	Sensitivity	Specificity	AUC	Avg_dif
D. Mitrea et al., 2022 [7]	97.79%	97.9%	98.9%	97.8%	−1.002
Aziz et al., 2021 [24]	84.77%	90%	79.4%	84.7%	**−14.38**
Paul et al., 2018 [27]	90.28%	94.1%	86.4%	95.9%	−7.43
Current approach	**98.9%**	**98.6%**	**99.2%**	**99.7%**	-

## Abbreviations

The following abbreviations are used in this manuscript:

HCC	Hepatocellular Carcinoma
PAR	Cirrhotic Parenchyma on which HCC had evolved
CNN	Convolutional Neural Networks
ML	Machine Learning
US	Ultrasonography
CT	Computer Tomography
MRI	Magnetic Resonance Imaging
AUC	Area under the ROC
FS	Feature Selection
RF	Random Forest
SVM	Support Vector Machines
SMO	The John Platt’s Sequential Minimal Optimization algorithm
PSO	Particle Swarm Optimization

## Appendix A

In Figure A1, the average standard deviations of the accuracy values for each dataset, GE7 and GE9, are depicted. The values for the average standard deviations resulted from computing the arithmetic mean of the standard deviations of the classification accuracies for each considered CNN for each considered fusion scheme.

## Appendix B

In Figure A2, the plots of the pairwise correlations between the textural features and the CNN features are depicted. In each plot, each feature is denoted by an index. In the case of the first dataset, GE7, the textural features have associated indexes from 1 to 550, while the CNN features have indexes from 551 to 2598 in the case of InceptionV3 and ResNet101, from 551 to 1788 in the case of EfficientNet_ASPP and from 551 to 1575 in the case of Convnext_base, respectively. In the case of the GE9 dataset, the textural features have associated indexes from 1 to 98, while the CNN features have indexes from 99 to 2047 in the case of InceptionV3 and ResNet101, from 99 to 1237 in the case of EfficientNet_ASPP and from 99 to 1122 in the case of Convnext_base, respectively. The fact that there exist high correlations (≥0.5) between the CNN features themselves can be noticed. There also exist correlations between the textural features and the CNN features but of smaller values (≤0.429). In Figure A2, the first column corresponds to the correlations derived from the first dataset, while the second column to the correlations computed on the second dataset; the first row corresponds to the correlations between the InceptionV3 features and the textural features, the second row to the correlations between the ResNet101 features and the textural features and the third row corresponds to the correlations between the EfficeintNetb0_ASPP features and the textural features, while the last row stands for the correlations between the the Convnext_base CNN features and the textural features.
